# Mnemonic-opto-synaptic transistor for in-sensor vision system

**DOI:** 10.1038/s41598-022-05944-y

**Published:** 2022-02-02

**Authors:** Joon-Kyu Han, Young-Woo Chung, Jaeho Sim, Ji-Man Yu, Geon-Beom Lee, Sang-Hyeon Kim, Yang-Kyu Choi

**Affiliations:** 1 School of Electrical Engineering, Korea Advanced Institute of Science and Technology, (KAIST) 291 Daehak-ro, Yuseong-gu, Daejeon, 34141 Republic of Korea; 2 Foundry Division, Samsung Electronics, Yongin, 17113 Republic of Korea

**Keywords:** Electrical and electronic engineering, Nanoscale devices

## Abstract

A mnemonic-opto-synaptic transistor (MOST) that has triple functions is demonstrated for an in-sensor vision system. It memorizes a photoresponsivity that corresponds to a synaptic weight as a memory cell, senses light as a photodetector, and performs weight updates as a synapse for machine vision with an artificial neural network (ANN). Herein the memory function added to a previous photodetecting device combined with a photodetector and a synapse provides a technical breakthrough for realizing in-sensor processing that is able to perform image sensing and signal processing in a sensor. A charge trap layer (CTL) was intercalated to gate dielectrics of a vertical pillar-shaped transistor for the memory function. Weight memorized in the CTL makes photoresponsivity tunable for real-time multiplication of the image with a memorized photoresponsivity matrix. Therefore, these multi-faceted features can allow in-sensor processing without external memory for the in-sensor vision system. In particular, the in-sensor vision system can enhance speed and energy efficiency compared to a conventional vision system due to the simultaneous preprocessing of massive data at sensor nodes prior to ANN nodes. Recognition of a simple pattern was demonstrated with full sets of the fabricated MOSTs. Furthermore, recognition of complex hand-written digits in the MNIST database was also demonstrated with software simulations.

## Introduction

The von Neumann architecture provides accurate calculations, however, it is not suitable for low power applications because of the data bottleneck between the memory and the processor^1^. In order to overcome the limitations of the von Neumann architecture, various artificial neuromorphic devices were explored to imitate functions of the brain. In details, two-terminal memristors such as resistive random-access memory (RRAM) and phase-change memory (PCM), and the three-terminal charge trap memory and electrochemical random-access memory (ECRAM) with separated reading and writing paths have been actively studied as synaptic devices for artificial neural networks (ANN)^2–6^.

**Figure 1:**
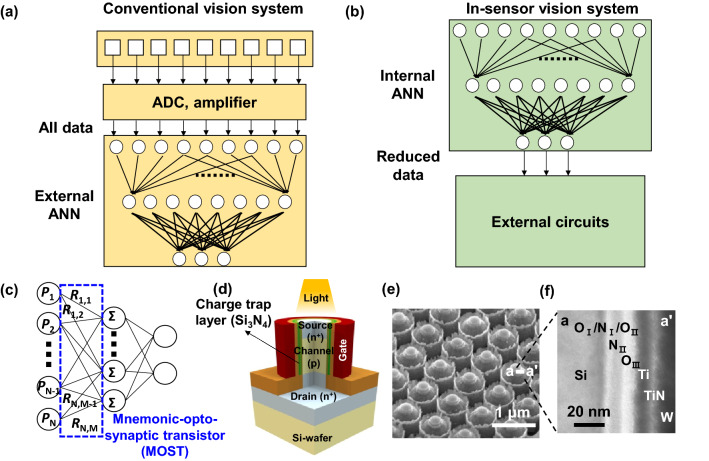
Schematic diagram of (**a**) conventional vision system and (**b**) in-sensor vision system. An internal artificial neural network (ANN) performs both sensing and preprocessing in a sensor for reduction of the signal latency and power consumption at converting circuits such as an analog-to-digital converter (ADC). (**c**) An internal ANN constituting an in-sensor vision system with the MOSTs, which can be located at the forefront of the ANN to simultaneously detect the optical signals and transmitting the preprocessed signal to the next layer. (**d**) Schematic of the mnemonic-opto-synaptic transistor (MOST). A charge trap layer (CTL) for tunable photoresponsivity and memory function is inserted into the gate dielectrics. (**e**) Scanning electron microscopy (SEM) image of the MOST array and (**f**) cross-sectional transmission electron microscopy (TEM) image of the gate region. Bandgap-engineered (BE) tunneling dielectrics (O_I_/N_I_/O_II_) were adopted to reduce the operating voltage.

By the way, vision systems assisted by neural processing allow accurate object detection, pattern recognition, and real-time image processing for robotics, autonomous vehicles, and sensory electronics^7–11^. A conventional vision system separates image sensing and signal processing. Its performance is thus adversely limited owing to signal latency and power consumption that arises from a huge amount of data processing with the inclusion of redundant data passing through a converting circuit such as an analog-to-digital converter (ADC), as illustrated in Fig. 1a^12–14^. In contrast, a biological retina performs sensing and simultaneous pre-processing of visual information in order to extract key features from the input visual data^15–18^. By the elimination of redundant visual data, subsequent information processing in the brain such as object detection and pattern recognition can become faster with lower power consumption.

Recently, inspired by a biological vision system, various optoelectronic synaptic devices that can act as both a photodetector and a synapse used for an ANN by preprocessing of the data in a sensor have been demonstrated^9–11^. During the optical sensing, however, their synaptic weight is changed owing to an optically controllable synaptic weight. This optical weight update is useful for recognizing one pattern or similar patterns, but it is difficult to recognize various subsequent patterns because the synaptic weights are customized to a previous pattern. Therefore, repetitive reset operations are needed before accepting new patterns. Unlike the abovementioned optoelectronic synaptic devices, Wang et al. and Mennal et al. demonstrated vision sensors where repetitive reset operations were unnecessary due to the invariant synaptic weight during the optical sensing. They reported tunable photoresponsivity using a photodetecting device composed of two-dimensional (2D) materials, such as a phototransistor or a photodiode^18,19^. The tunable photoresponsivity in a photodetecting device corresponds to the controllability of weight update in a synapse, and it is a significant advantage for an in-sensor vision system, because photoresponsivity tunable photodetecting device can act as a synapse for an ANN as well as a photodetector for a sensor. Thus, the in-sensor processing with the inclusion of image sensing and signal processing allows real-time multiplication of the image with a memorized photoresponsivity matrix. Such an in-sensor vision system is attractive for reduction of signal latency and power consumption, which occur at converting circuits such as the ADC, as illustrated in Fig. 1b.

It is worth noting that the previous photodetecting device with tunable photoresponsivity requires external memory, which is indispensable for storing the value of gate voltage to tune the photoresponsivity^18,19^. This memory can impose a burden on accessing a designated memory cell with high speed and realizing a mobile vision system with a compact size for an all-in-one chip. Thus, signal latency and power consumption that arise from external memory become increasingly problematic. In addition, 2D materials cannot be easily integrated by microfabrication of a complementary metal–oxide–semiconductor (CMOS) based image sensor system with high throughput owing to less CMOS compatibility. For a large-scale vision system, a CMOS compatible photodetecting device such as a photodiode and a phototransistor is preferred; however, tunable photoresponsivity is not available. Each approach for tunable photoresponsivity without CMOS compatibility and CMOS compatibility without tunable photoresponsivity has its respective strengths and weaknesses. Therefore, it is very timely to explore another photodetecting device with tunable photoresponsivity, CMOS compatibility, and even more memorability.

In this work, a mnemonic-opto-synaptic transistor (MOST) is demonstrated in the form of a metal–oxide–semiconductor field-effect transistor (MOSFET). This MOSFET has a vertical pillar-shaped channel protruded from a silicon bulk substrate and a gate wraps a sidewall of the pillared channel completely with a gate-all-around structure. This vertical MOSFET is advantageous from the perspective of the footprint area and light absorption^20–22^. Moreover, by embedding a charge trap layer (CTL) of a nitride (Si_3_N_4_) to the gate dielectrics of the MOST for the memory function, individual control of photoresponsivity for each MOST is achieved and real-time multiplication of the image with a memorized photoresponsivity matrix is performed. Therefore, it can act as a photodetector and a synapse with non-volatile retention of learned weights in the ANN for the in-sensor vision system due to the intrinsic memory function of the intercalated CTL. It does not need repetitive reset operations because the synaptic weight is not changed during the optical sensing. This characteristic is attributed to fully electrical control of the synaptic weight. Furthermore, by virtue of 100% CMOS compatible fabrication, it can be integrated with a conventional large-scale CMOS image sensor system comprising numerous small-sized pixels. After optical and electrical characterization of the MOST, recognition of a simple pattern is performed using the sets of the fabricated devices, and recognition of a complex MNIST hand-written number is exploited using software simulations.

## Results and discussion

Figure 1c represents the ANN for the in-sensor vision system using the MOSTs. The MOSTs are located at the forefront of the ANN for detecting the light intensity and transmitting pre-processed weights with a reflection of optical signals to the next layer. The photocurrent (*I*_photo_) summed from each neuron at the next layer is produced by the multiplication of the memorized photoresponsivity matrix and the light intensity of each pixel. When the vision system has *N* pixels and *M* neurons at the next layer, current summed in the *m*th neuron of the next layer (*I*_m_) can be represented by the following equation: ${I}_{m}=\sum_{n=1}^{N}{I}_{photo}=\sum_{n=1}^{N}{R}_{mn}{P}_{n}$, where *n* = 1, 2, …, *N* and *m* = 1, 2, …, *M* denote the indices of the pixel and the neuron at the next layer, respectively. *R*_mn_ represents the memorized photoresponsivity matrix and *P*_n_ represents the light intensity of each pixel. In this way, the in-sensor processing with the inclusion of image sensing and signal processing allows real-time multiplication of the image with the memorized photoresponsivity matrix^19^.

Figure 1d shows a schematic of an n-channel MOST with a vertical pillar structure. n^+^ heavily doped source (S) and drain (D) are located at the top and the bottom of each pillar in the array of MOSTs shown in Fig. 1e, which protrudes from a bulk-silicon wafer, respectively. Between the S and D, there is a p-type channel. As gate dielectrics, quintuple-layers (O_I_/N_I_/O_II_/N_II_/O_III_) composed of triple-layered tunneling dielectrics (O_I_/N_I_/O_II_), the aforementioned CTL nitride (N_II_), and a blocking oxide (O_III_) wrap around a sidewall of the pillared channel, as shown in Fig. 1f. The triple layers of the O_I_/N_I_/O_II_ were adopted to reduce the operating voltage by barrier engineering (BE) of the tunneling dielectrics^23,24^. Each thickness of the gate dielectrics is 1.3 nm/1.3 nm/1.6 nm/5.6 nm/6.3 nm in the order of O_I_/N_I_/O_II_/N_II_/O_III_, respectively. A triple-layered metal gate composed of titanium, titanium nitride, and tungsten (Ti/TiN/W) also surrounds the sidewall exterior of the gate dielectrics and pillar. When the light is illuminated, the carriers are generated and flown in the channel in the form of *I*_photo_ that drives the photodetector. *I*_photo_ is actually the drain current (*I*_D_) flowing between the source and the drain, which is controlled by the gate voltage (*V*_G_) and drain voltage (*V*_D_). The gate electrode makes the photoresponsivity tunable by charging and discharging the CTL of N_II_ (hereafter simply abbreviated as ‘CTL’) and controls the memory function. Note that N_I_ in the tunneling dielectrics cannot serve as a CTL because O_I_ is too thin to block tunneling of the trapped charges. Fabrication details of the MOST are described in Figure S1.

**Figure 2:**
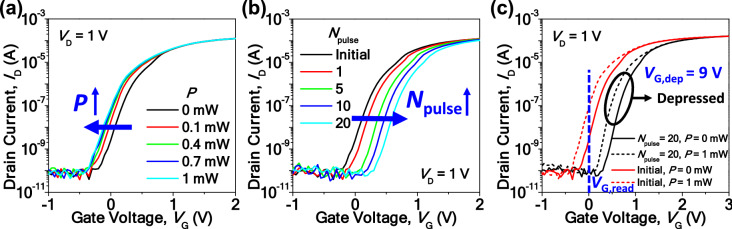
(**a**) Transfer characteristics (*I*_D_–*V*_G_) of the MOST for various light intensities (*P*). Leftward *V*_T_ shift with increased *P* that corresponds to a temporal response by the photo-carrier density. (**b**) *I*_D_–*V*_G_ for various *N*_pulse_ with + *V*_G_. Rightward *V*_T_ shift with increased *N*_pulse_ that corresponds to a semi-permanent response by the trapped electron density. This is a depression operation for reducing the weight of the synaptic device. (**c**) *I*_D_–*V*_G_ at dark and 1 mW light illumination before and after depression. Photocurrent (*I*_photo_) at read gate voltage (*V*_G,read_) of 0 V is approximately 0.1 μA before the depression and 0.1 nA after the depression, respectively. In this way, the trapped electron density tunes the photoresponsivity.

In the MOST, threshold voltage (*V*_T_) can be adjusted by two factors, photo-carriers controlled by light illumination and trapped electrons modulated by the *V*_G_ in the CTL. Figure 2 shows the transfer characteristic curve of *I*_D_ versus *V*_G_ (*I*_D_–*V*_G_) according to the light intensity (*P*) and the number of gate pulses (*N*_pulse_). This *N*_pulse_ determines the level of *I*_D_ at each state in the synaptic operation, i.e., the number of states. As an example, *N*_pulse_ of 0 is the initial state with the highest *I*_D_ due to the lowest *V*_T_, and *N*_pulse_ of 31 is composed of 31 gate pulses that produce the lowest *I*_D_ due to the highest *V*_T_ in the depression for multi-states of 32. In this work, a variable pulse number with an identical pulse amplitude and width is used for a potentiation–depression (P–D) operation. An LED (SOL 3.0, Fiber Optic Korea Co., Ltd.) was used as a white light source. The *P* indicated in Fig. 2 is the measured value in a blue region with a wavelength of 405 nm. It was quantified by a power meter that has a detection spot area of 0.785 cm^2^. Figure 2a shows a leftward *V*_T_ shift. This is caused by the photo-carrier generation, which arises from light illumination^25^. In contrast, Fig. 2b exhibits a rightward *V*_T_ shift. It is attributed to electron trapping in the CTL by applied positive depression gate voltage (*V*_G,dep_); i.e., it suppresses inversion at the channel surface. This is analogous to the depression operation to reduce the synaptic weight in an artificial synapse^26–28^. The magnitude of *V*_G,dep_ is 9 V and its pulse width is 10 μs. It should be noted that the rightward *V*_T_ shift by the electron trapping is semi-permanent and the leftward *V*_T_ shift by the light illumination is temporal. In other words, the *V*_T_ shift is returned to a pristine state when the light illumination is removed. Figure 2c superimposes *I*_D_–*V*_G_ with the photo-carrier generation by incident light and the electron trapping by the applied *V*_G,dep_ in one graph. The ratio (*η*) of photoresponsivity without charge trapping to that with charge trapping by *V*_G_ is approximately 800 at a *V*_G,read_ of 0 V. In this way, photoresponsivity can be modulated effectively by controlling the trapped electrons in the CTL. Therefore, the MOST acts as a photodetector by sensing *I*_photo_ with light, a synapse by updating a weight with *V*_G_, and a non-volatile memory by holding a weighted state with trapped charges for the in-sensor vision system. This tunable photoresponsivity is utilized as a controllable synaptic weight in the ANN. Unlike the previously reported photodetecting device, extra memory is no longer needed because the MOST itself harnesses an inherent non-volatile memory function^18,19^.

**Figure 3:**
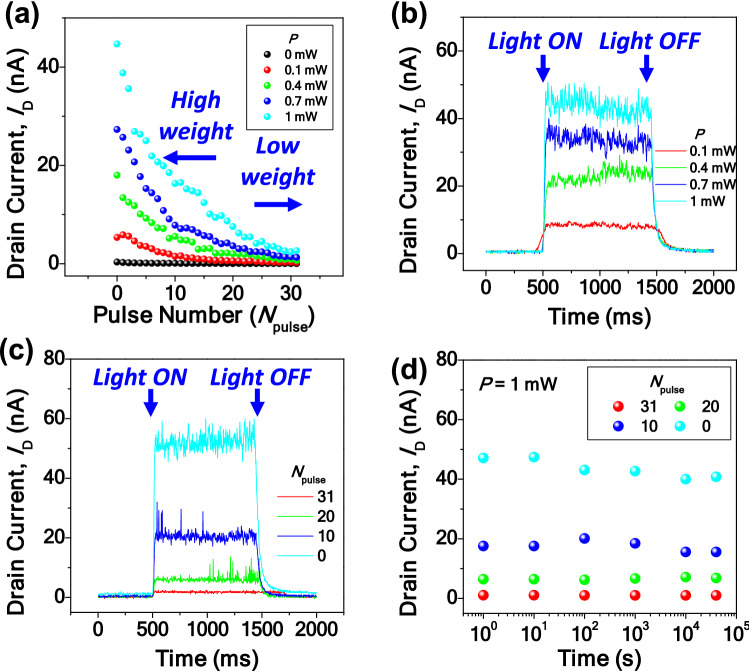
(**a**) *I*_D_ versus *N*_pulse_ for various *P*. The photoresponsivity is reduced by the depression. (**b**) Real-time *I*_D_ for various *P* when *N*_pulse_ is zero. *I*_D_ is increased as *P* increases. (**c**) Real-time *I*_D_ for various *N*_pulse_ when *P* is 1 mW. *I*_D_ is decreased as *N*_pulse_ increases. (**d**) Retention characteristics of the MOST for various *N*_pulse_ when *P* is 1 mW.

Figure 3a shows the depression where *I*_D_ was decreased by an increased *N*_pulse_ for various *P*. Herein *N*_pulse_ is varied from 0 to 31; i.e., there are 32 states. The magnitude of *V*_G,dep_ is 9 V and its pulse width is 1 μs. This result shows that the photoresponsivity was finely tunable with multi-states. For a typical synaptic operation, the potentiation that increases the synaptic weight should be available, similar to the depression that decreases the synaptic weight. Figure S2(a) represents the P-D characteristics for various *P*, i.e., with light illumination. The conductance (*G*) is defined as *I*_D_/*V*_D_, which can be simplified to *I*_D_ because the applied *V*_D_ was 1 V. The photoresponsivity was finely tunable during the potentiation as well as the depression. The magnitude of potentiation gate voltage (*V*_G,pot_) is − 10 V and its pulse width is 200 μs. Figure S2(b) shows another P-D characteristic in a dark environment, i.e., without light illumination. From Figure S2(b), the nonlinearity parameters (*α*) were extracted using the following equation:

1 $$ G = \left\{ {\begin{array}{l} {\left( {\left( {G_{max}^{\alpha } - G_{min}^{\alpha } } \right) \times w + G_{min}^{\alpha } } \right)^{1/\alpha } \quad if \alpha \ne 0,} \\ {G_{min}^{\alpha } \times (G_{max} /G_{min} )^{w} \quad if \alpha = 0.} \\ \end{array} } \right. $$

where *G*_max_ is the maximum conductance, *G*_min_ is the minimum conductance, *α* is a nonlinear parameter, and *w* is an internal variable that ranges from 0 to 1^29^. The extracted *α*_pot_ and *α*_dep_ were − 0.02 and − 0.58, respectively. These parameters are used for the subsequent software simulations. It is well known that a large number of states is preferred to enhance the performance of pattern recognition in a synaptic device^26–28^. In this context, it was also confirmed that the P–D characteristics for *N*_pulse_ of 64 and 128 were achievable by delicately tuning the gate pulse, as shown in Figure S3.

Figure 3b, c show the real-time *I*_D_ for various *P* and *N*_pulse_, respectively, when the light is turned on and off. At a fixed *N*_pulse_, *I*_D_ was increased as *P* increased. At a fixed *P*, *I*_D_ decreased as the *N*_pulse_ increased. It is worth noting that *I*_D_ returned to the initial state when the light was off. This feature assures that the synaptic weight is not changed during the optical sensing and repetitive reset operations are not needed. As shown in Fig. 3d, *I*_D_ was sustained even after 40,000 s owing to the superior retention characteristics of the CTL-based memory. This attribution has been proven by commercial flash memory adopting the CTL. It should be recalled that good retention characteristics of a synaptic device are crucial for reliable operation over time^28^.

Figure S4 shows the P–D characteristics of the MOST for various wavelengths (*λ*). Measurements were performed by using a blue (B), red (R), and infrared (IR) light source. Each *λ* of B, R, and IR light is 405 nm, 638 nm, and 1550 nm, respectively. As shown in Figure S4, tunable photoresponsivity was observed for visible light of B and R, whereas it was not for the IR light. This is because the B and R light can generate photo-carriers to increase *I*_photo_. However, the IR light cannot create them owing to a small photon energy of 0.80 eV compared to the silicon energy bandgap of 1.12 eV^30,31^. It should also be noted that the photoresponsivity of the B light was smaller than that of the R light because the penetration depth is decreased with shorter *λ*^32^. The demonstrated wavelength dependency as well as the intensity dependency of the tunable photoresponsivity can help in recognizing a color mixed pattern^33,34^.

As mentioned above, BE tunneling dielectrics composed of the triple layers renamed BE layers were adopted to reduce the operating voltage. In order to confirm this effect, simplified MOSTs were fabricated as a control group. The BE layers of O_I_/N_I_/O_II_ were replaced by a single layer of thermal oxide (O_single_). Other structures were set to be the same. As plotted in Figure S5(a), the measured transfer characteristics of the fabricated MOST with O_single_/N_II_/O_III_ showed similar photoresponsivity compared to those with O_I_/N_I_/O_II_/N_II_/O_III_. This is because the gate dielectric has no effect on the photo-carrier generation by light. Whereas *V*_T_ was shifted rightward by a *V*_G,dep_ of 9 V in the case of the O_I_/N_I_/O_II_/N_II_/O_III_ (Fig. 2), it was not changed by that in the case of the O_single_/N_II_/O_III_, as shown in Figure S5(b). A *V*_G,dep_ larger than 11 V should be applied to change the *V*_T_ and update the synaptic weight, as shown in Figure S5b. As a consequence, the P–D characteristics in Figure S5(c) show that synaptic weight update is impossible with the same *V*_G,dep_ in the case of the O_single_/N_II_/O_III_. Therefore, it is confirmed that the gate dielectric structure of O_I_/N_I_/O_II_/N_II_/O_III_ is more attractive than that of O_single_/N_II_/O_III_ for low-power neuromorphic hardware.

**Figure 4:**
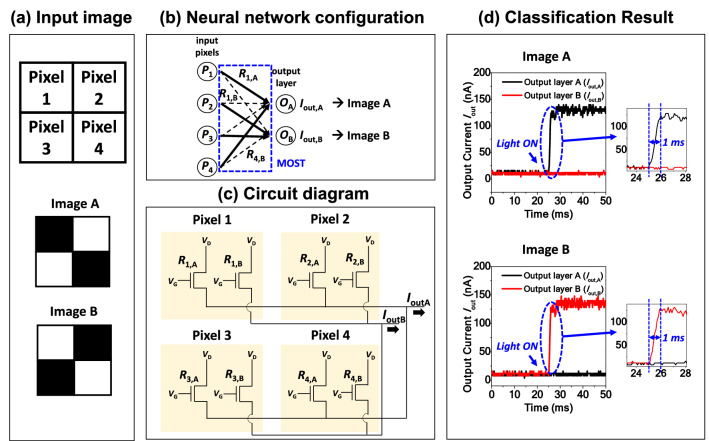
Demonstration of hardware-based pattern recognition. (**a**) Two input images ‘A’ (off-diagonal) and ‘B’ (diagonal), which are composed of 2 × 2 black-and-white pixels. (**b**) Neural network and (**c**) Circuit diagram for the 2 × 2 pattern recognition. They are composed of eight MOSTs with each tunable photoresponsivity represented as ‘*R*’. (**d**) Measured classification data of the off-diagonal and diagonal patterns. By comparing two output currents (*I*_out,A_ and *I*_out,B_), fast classification within 1 ms was achieved with low power consumption under 150 nW.

Using a full set of the fabricated MOSTs, simple pattern recognition was performed using a single-layer perceptron (SLP). As illustrated in Fig. 4a, two images, ‘A’ of an off-diagonal pattern and ‘B’ of a diagonal pattern, were prepared. Each pattern comprises 2 × 2 black-and-white pixels. Classification of the two patterns was attempted. A neural network was composed of four input pixels labeled *P*_1_, *P*_2_, *P*_3_, and *P*_4_ and two nodes in the output layer labeled *O*_A_ and *O*_B_, as depicted in Fig. 4b. By detecting the output current of the MOSTs connected to each output node, each pattern was recognized. The photoresponsivity that corresponds to the synaptic weight was preset with a binary value, the maximum photoresponsivity and the minimum photoresponsivity, from the data of Fig. 3a. The solid lines and the dashed lines in Fig. 4b represent the device with the maximum photoresponsivity and the minimum photoresponsivity, respectively. Each photoresponsivity is represented as ‘*R*’ in the neural network configuration. This in-sensor processing with the inclusion of image sensing and signal processing performs real-time multiplication of the image with a memorized photoresponsivity matrix^19^. Figure 4c shows the circuit diagram to construct the neural network of Fig. 4b. *V*_G_ and *V*_D_ were set as 0 V and 1 V, respectively. Each output was measured in the form of the output current: *I*_out,A_ and *I*_out,B_; i.e., *I*_out,A_ was measured in the output node *O*_A_ for the input image of ‘A’ and *I*_out,B_ was measured in the output node *O*_B_ for the input image of ‘B’, as shown in Fig. 4d. As a result, inference for the simple pattern was experimentally verified. It is worth comparing the required components to distinguish the abovementioned two simple patterns. This work that is applicable to an in-sensor vision system demands only eight MOSTs without extra photodetectors, ADCs or synaptic devices. In contrast, a conventional approach that is suitable for a conventional vision system may need four photodetectors, an ADC, and eight synaptic devices. Thanks to this in-sensor vision system, rapid classification within 1 ms was achieved with low power consumption under 150 nW. This is very small compared to the power consumption of an ADC used for a conventional vision system, which ranges from a few tens of μW to a few mW^35,36^.

**Figure 5:**
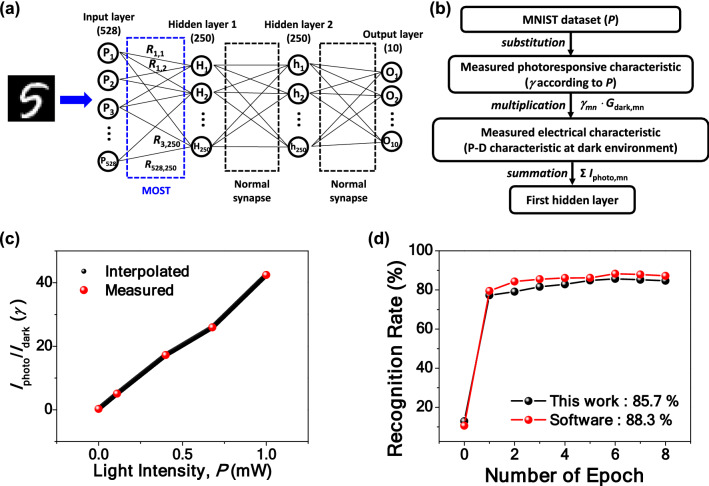
Demonstration of software-based pattern recognition. (**a**) Neural network for recognition of hand-written numbers in the MNIST dataset. Photoresponsive optical characteristics (sensory function) and non-volatile electrical characteristics (mnemonic and synaptic function) measured from the fabricated MOSTs are reflected at the forefront of the network. Measured electrical characteristics are reflected in normal synapses that are connected to the first hidden layer and the second hidden layer, or the second hidden layer and the output layer. (**b**) Software-based simulation sequence to reflect the measured characteristics of the MOST. (**c**) *γ* (≡*I*_photo_/*I*_dark_) as a function of the light intensity (*P*). Linear interpolation from the measured data is utilized to create extra data. (**d**) Simulated recognition accuracy according to the number of training epochs. Recognition rate of 85.7% is achieved, which is close to the upper limit of 88.3% by an ideal software-based algorithm.

To demonstrate recognition of more complex patterns such as hand-written digits in the MNIST dataset, a multi-layer perceptron (MLP) network composed of two hidden layers was constructed, as illustrated in Fig. 5a. An input layer corresponds to 528 input pixels, which were cropped from the 28 × 28 pixels, and an output layer corresponds to the 10 numbers from 0 to 9. Each hidden layer is composed of 250 neurons. The MOSTs were located at the forefront of the network for detecting the light intensity and transmitting pre-processed weights with a reflection of optical signals to the first hidden layer. Each device has its own photoresponsivity corresponding to the synaptic weight, which is represented as ‘*R*’ in the neural network configuration. This simultaneous image sensing and signal processing allow real-time multiplication of the image with a memorized photoresponsivity matrix^19^. The measured photoresponsive and P-D characteristics from the fabricated MOSTs in a dark environment were reflected in the software simulations. Figure 5b shows a flow chart that summarizes the simulation sequence to reflect the measured photoresponse characteristics and electrical characteristics of the fabricated MOST. *I*_photo_ is the drain current with light illumination (*I*_D,light_) and *I*_dark_ is the referenced drain current without light illumination (*I*_D,dark_). Except light-on and light-off, all other conditions are the same. Herein the ratio of *I*_photo_/*I*_dark_, i.e., *I*_D,light_/*I*_D,dark_ is defined as *γ*, which is extracted from the experimental results. Prior to the simulation, *γ* was extracted for various light intensities (*P*) by linear interpolation, as shown in Fig. 5c. For improvement of the simulation accuracy, this step was repeated for each synaptic state. *γ* of each pixel was extracted by substituting the MNIST dataset into the interpolated curve, because the MNIST dataset represents the pixel intensity. Afterwards, the conductance of each synapse in a dark environment (*G*_dark_), which was extracted from the P–D characteristic of Figure S2(b), was multiplied by *γ*. Because the applied *V*_D_ of the MOST is 1 V, *G*_dark_, defined as *I*_dark_/*V*_D_, is simplified to the *I*_dark_. The multiplication thus results in *I*_photo_. Finally, *I*_photo_ that contains information of the pixel intensity and the photoresponsivity of the synapse is transmitted to the first hidden layer for summation at each neuron. In detail, current summed in the *m*th neuron in the first hidden layer (*I*_m_) can be represented by the following equation:

$$ I_{m} = \mathop \sum \limits_{n = 1}^{528} I_{photo,mn} = \mathop \sum \limits_{n = 1}^{528}\gamma_{mn} G_{dark,mn}, $$

where *n* = 1, 2, …, 528 and *m* = 1, 2, …, 250 denote the indices of the pixel and the neuron at the first hidden layer, respectively.

For a normal synapse between the first hidden layer and the second hidden layer or between the second hidden layer and the output layer, only the electrical characteristics (e.g., P–D characteristics at dark environment) were reflected because they could not respond to the light owing to deficiency of a photo-effect. The sigmoid activation function was adopted and supervised learning with back propagation was employed for the learning process to update the synaptic weight of the MOST and a normal synapse. Figure 5d shows the simulated recognition accuracy according to the number of training epochs and the saturated recognition rate was 85.7%. This recognition rate is comparable to an upper limit of 88.3%, which is achievable by software-based pattern recognition simulations that directly multiply the MNIST dataset by the conductance of each synapse, which has ideal P-D characteristics of perfect linearity and symmetry; i.e., *α*_pot_ = 1 and *α*_dep_ = 1.

## Conclusions

In summary, a mnemonic-opto-synaptic transistor (MOST) was demonstrated for an in-sensor vision system by embedding a non-volatile memory function into a photodetecting device. Because the threshold voltage of the MOST was controlled both by light illumination and by an electrical pulse, the photoresponsivity was tunable by changing the trapped electrons in the charge trap layer (CTL) that enable the non-volatile memory function. Thereby it performed triple functions: photoresponsivity memorizing as a memory cell, light-sensing as a photodetector, and weight updating as a synapse. At the forefront of the ANN, the MOST simultaneously detects light and generates a pre-processed signal to perform real-time multiplication of an image with a memorized photoresponsivity matrix in sensors. More advantageously, it does not require repetitive reset operations because of the invariant synaptic weight during the optical sensing (Table S6). Furthermore, it does not require external memory because of the inherent memory function of the CTL. In addition, the MOST can be integrated with a conventional CMOS image sensor composed of numerous small-sized pixels because it was fabricated with 100% CMOS compatible microfabrication.

## Supplementary Information

Supplementary Information.

## Data Availability

*Scientific Reports* requires the inclusion of a data availability statement with all submitted manuscripts, as this journal requires authors to make available materials, data, and associated protocols to readers.
