# Supplementary material for: Mnemonic-opto-synaptic transistor for in-sensor vision system
Source: Sci Rep. 2022 Feb 2;12:1818. doi: 10.1038/s41598-022-05944-y (PMC8810857; doi:10.1038/s41598-022-05944-y)
Supplement: Supplementary file 1 — Supplementary Information. [file 41598_2022_5944_MOESM1_ESM.docx]

**Supplementary Information**

Mnemonic-Opto-Synaptic Transistor for In-sensor Vision System

Joon-Kyu Han^1†^, Young-Woo Chung^1,2†^, Jaeho Sim^1^, Ji-Man Yu^1^, Geon-Beom Lee^1^, Sang-Hyeon Kim^1^ and Yang-Kyu Choi^1*^

^1^ School of Electrical Engineering, Korea Advanced Institute of Science and Technology, (KAIST) 291 Daehak-ro, Yuseong-gu, Daejeon 34141, Republic of Korea
^2^ Foundry Division, Samsung Electronics, Yongin 17113, Republic of Korea
^*^ykchoi@ee.kaist.ac.kr

^⸸^these authors contributed equally to this work

**1. Fabrication process**

The fabrication procedure of the mnemonic-opto-synaptic transistor (MOST) is shown in Figure S1. First, as illustrated in Figure S1(a), three-step iterative ion implantations, deep phosphorus (P) implantation for the drain, medium boron (B) implantation for the body, and shallow arsenic (As) implantation for the source, were performed on a bulk-Si wafer. Then, as shown in Figure S1(b), the vertical pillar was formed by photo-lithography and dry etching. The 1^st^ inter-layer dielectric (ILD) was then deposited for device-to-device isolation. Afterwards, the bandgap-engineered (BE) tunneling layers composed of silicon oxide (O_Ⅱ_), silicon nitride (N_Ⅰ_), and silicon oxide (O_Ⅱ_) were formed sequentially. Another silicon nitride (N_Ⅱ_) layer that acts as a charge trap layer and another silicon oxide (O_Ⅲ_) layer that acts as a blocking layer were deposited. Afterwards, titanium (Ti), titanium nitride (TiN), and tungsten (W) that serve as a gate electrode metal (W) were sequentially deposited. They were then etched until the top of the source in the Si-pillar was exposed; *i*.*e*., they were left at the sidewall of the Si-pillar, as illustrated in Figure S1(c). After the 2^nd^ ILD deposition, contact holes were opened and Ti/TiN/W were again deposited and patterned for the metal interconnections, as shown in Figure S1(d). Finally, the vertical pillar-typed MOST was fabricated with a pillar diameter (*D*_pillar_) of 320 nm and a pillar height (*H*_pillar_) of 700 nm.

~~~~

**Figure S1.** Fabrication procedure of the MOST. (a) Three-step iterative ion implantations for a drain, a channel, and a source. (b) Vertical pillar formation and 1^st^ inter-layer-dielectric (ILD) deposition. (c) Formation of bandgap-engineered (BE) tunneling layers (O_Ⅰ_/N_Ⅰ_/O_Ⅱ_), charge trap layer (N_Ⅱ_), blocking layer

**2. Potentiation-depression (P-D) characteristics**

From the fabricated MOST, the P-D characteristics were characterized, as shown in Figure S2. These characteristics have widely been used to confirm the synaptic operation, in which the conductance (weight) is changeable according to the applied pulse number (*N*_pulse_).^1-3^ For the potentiation, a gate pulse with a magnitude of -10 V and a width of 200 μs was used. For the depression, a gate pulse with a magnitude of 9 V and a width of 1 μs was used. Figure S2(a) shows the P-D characteristics at various light intensities. By controlling the synaptic weight, the photoresponsivity is tunable. For example, the conductance at 1 mW light illumination is 44.7 nS after full potentiation, while that at 1 mW light illumination is 2.25 nS after full depression. Figure S2(b) shows the P-D characteristic at a dark environment. The nonlinearity parameters (*α*) were extracted: -0.02 for potentiation (*α*_pot_) and -0.58 for depression (*α*_dep_). These parameters were used for the software-based simulations.

**Figure S2.** (a) Potentiation-depression (P-D) characteristics at various light intensities. (b) P-D characteristic at a dark environment. The extracted *α*_pot_ and *α*_dep_ were -0.02 and -0.58, respectively.

**3. Potentiation-depression (P-D) characteristics with various number of states (*N*_bit_)**

It is well known that the number of synaptic states (*N*_bit_) should be increased to enhance the performance of pattern recognition.^1-3^ As shown in Figure 3, *N*_bit_ is increased by reducing the gate pulse width. For 32 states (=2^5^; *N*_bit_=5), the gate pulse width for the depression and the potentiation was 1 μs and 0.2 ms, respectively. For 64 states (=2^6^; *N*_bit_=6), the gate pulse width for the depression and the potentiation was 0.5 μs and 0.12 ms, respectively. For 128 states (=2^7^; *N*_bit_=7), the gate pulse width for the depression and the potentiation was 0.33 μs and 0.1 ms, respectively.

**Figure S3.** P-D characteristics for 64 and 128 states. (a) 64 states (=2^6^; *N*_bit_=6) at various light intensities and (b) at a dark environment. The extracted *α*_pot_ and *α*_dep_ were 0.05 and -1.28, respectively. (c) 128 states (=2^7^; *N*_bit_=7) at various light intensities and (d) at a dark environment. Extracted *α*_pot_ and *α*_dep_ were 0.49 and -0.19, respectively.

**4. Wavelength dependency**

For recognition of a color mixed pattern, it is important to investigate how the wavelength (*λ*) of the illuminated light can influence the conductance change.^4,5^ Three light sources with different *λ*, blue light (B) of *λ*=405 nm, red light (R) of *λ*=638 nm and infrared light (IR) of *λ*=1550 nm, were used. As shown in Figure S4, the photoresponse was not observed in the case of the IR light because the IR light cannot generate carriers in the channel due to the smaller photon energy compared to the silicon energy bandgap. The photoresponse of B-light was smaller than that of R-light because the penetration depth decreases for shorter *λ*. For all the experiments with IR-, R-, and B-light, each intensity was 3 μW.

**Figure S4.** P-D characteristics of 32 states depending on the wavelength of the illuminated light. Blue (B), red (R), and infrared (IR) light sources were used.

**5. Effect of bandgap engineered (BE) tunneling layer**

As an experimental group, MOSTs composed of triple tunneling layers (O_Ⅰ_/N_Ⅰ_/O_Ⅱ_) were fabricated. As a control group, MOSTs comprising a single tunneling layer (O_single_) were also fabricated. As shown in Figure S5(a), typical transfer characteristics and photoresponsive characteristics were confirmed in the control group. Unlike the MOST with O_Ⅰ_/N_Ⅰ_/O_Ⅱ_, *V*_T_ was not shifted with a pulse magnitude of 9 V in the MOST with O_single_, as shown in Figure S5(b). As a result, the P-D characteristics in Figure S5(c) show that synaptic weight update is not available with the same pulse conditions applied to the MOST with O_Ⅰ_/N_Ⅰ_/O_Ⅱ_. Note that a gate pulse larger than 11 V should be applied to induce a *V*_T_ shift and to update the synaptic weight in the case of the MOST with O_single_.

**Figure S5.** (a) Transfer characteristics (*I*_D_-*V*_G_) of the MOST composed of O_single_ as the tunneling layer (control group). (b) *I*_D_-*V*_G_ for various gate voltages (*V*_G,dep_) with 10 μs pulse width. (c) Comparison of the P-D characteristics at a dark environment for 32 states between the control and experimental group. Higher gate voltage is needed to update the synaptic weight in the control group (O_single_) compared to the experimental group (O_Ⅰ_/N_Ⅰ_/O_Ⅱ_).

**6. Comparison table with previous works**

Table S6 shows the comparison table with previous works and this work to report various kinds of a photodevice used for the ANN. In this work, repetitive reset operations are not necessary because the synaptic weight is not changed during the optical sensing. This is attributed to the electrical control of both photosensitive potentiation and depression. In addition, an external memory is not necessary owing to the internal memory function due to the charge trapping in the CTL of the MOST. It can be easily integrated into a conventional CMOS image sensor (CIS) due to its full CMOS compatibility.

**Table S6.** Comparison table between previous works and this work. The weight is not changed during optical sensing, and an external memory is not required thanks to the internal memory function. In addition, the MOST was fabricated with 100 % CMOS compatible microfabrication.

**Supporting information reference**

1. Seo, M. *et al*. First Demonstration of a Logic-Process Compatible Junctionless Ferroelectric FinFET Synapse for Neuromorphic Applications. *IEEE Electron Device Lett.* **39**, 1445− 1448 (2018).

2. Yu, J.-M. *et al*. All-Solid-State Ion Synaptic Transistor for Wafer-Scale Integration with Electrolyte of a Nanoscale Thickness. *Adv. Funct. Mater*. **31**, 2010971 (2021).

3. Yu, S. Neuro-Inspired Computing with Emerging Nonvolatile Memory, *Proc. IEEE.* **106**, 260-285 (2018).

4. Seo, S. *et al*. Artificial Optic-Neural Synapse for Colored and Color-Mixed Pattern Recognition. *Nat. Commun.* **9**, 5106 (2018).

5. Kim, S. K. *et al*. Photo-Responsible Synapse Using Ge Synaptic Transistors and GaAs Photodetectors. *IEEE Electron Device Lett.* **41**, 605−608 (2020).

6. Zhou, F. *et al*. Optoelectronic Resistive Random Access Memory for Neuromorphic Vision Sensors. *Nat. Nanotechnol.***14**, 776−782 (2019).

7. Qiu, W. *et al*. Optoelectronic In‐Ga‐Zn‐O Memtransistors for Artificial Vision System. *Adv. Funct. Mater*. **30**, 2002325 (2020).

8. Ahmed, T. *et al*. Fully Light‐Controlled Memory and Neuromorphic Computation in Layered Black Phosphorus. *Advanced Materials.* **33**, 2004207 (2021).

9. Zhai, Y. *et al*. Near infrared neuromorphic computing via upconversion-mediated optogenetics. *Nano Energy.* **67**, 104262 (2020).

10. Wang, C. Y. *et al*. Gate Tunable Van Der Waals Heterostructure for Reconfigurable Neural Network Vision Sensor. *Sci. Adv*. **6**, eaba6173 (2020).

11. Mennel, L. *et al*. Ultrafast Machine Vision with 2D Material Neural Network Image Sensors. *Nature.* **579**, 62−66 (2020).
